# Clinical subtyping using community detection: Limited utility?

**DOI:** 10.1002/mpr.1951

**Published:** 2022-11-22

**Authors:** Joost A. Agelink van Rentergem, Joe Bathelt, Hilde M. Geurts

**Affiliations:** ^1^ Department of Psychology Dutch Autism & ADHD Research Centre (d’Arc) University of Amsterdam Amsterdam The Netherlands; ^2^ Department of Psychology Royal Holloway University of London Egham UK; ^3^ Leo Kannerhuis (Youz/Parnassia Groep) Amsterdam The Netherlands

**Keywords:** community detection, hierarchical clustering, k‐means, latent profile analysis, psychiatric subtypes

## Abstract

**Objectives:**

To discover psychiatric subtypes, researchers are adopting a method called community detection. This method was not subjected to the same scrutiny in the psychiatric literature as traditional clustering methods. Furthermore, many community detection algorithms have been developed without psychiatric sample sizes and variable numbers in mind. We aim to provide clarity to researchers on the utility of this method.

**Methods:**

We provide an introduction to community detection algorithms, specifically describing the crucial differences between correlation‐based and distance‐based community detection. We compare community detection results to results of traditional methods in a simulation study representing typical psychiatry settings, using three conceptualizations of how subtypes might differ.

**Results:**

We discovered that the number of recovered subgroups was often incorrect with several community detection algorithms. Correlation‐based community detection fared better than distance‐based community detection, and performed relatively well with smaller sample sizes. Latent profile analysis was more consistent in recovering subtypes. Whether methods were successful depended on how differences were introduced.

**Conclusions:**

Traditional methods like latent profile analysis remain reasonable choices. Furthermore, results depend on assumptions and theoretical choices underlying subtyping analyses, which researchers need to consider before drawing conclusions on subtypes. Employing multiple subtyping methods to establish method dependency is recommended.

## 1 INTRODUCTION

Researchers in psychiatry are increasingly adopting data analysis methods from network science (Robinaugh et al., 2020). Networks are becoming popular to describe the complex dynamics present in psychiatry (Bringmann et al., 2015). Networks can be used to describe any set of relations between entities and variables. These entities or variables are referred to as nodes. To find out whether specific nodes within a network cluster together, community detection was developed (Fortunato, 2010), which provides an alternative to clustering and latent profile methods that are familiar to psychiatric researchers. A community in the present article refers to a subgroup^1^ of people from a larger population that is not a priori defined by any known variable. The problem is that while community detection methods are easy to apply, the properties of community detection have not been studied as extensively for psychological test score applications as for other applications (Gates et al., 2014, 2019; Lancichinetti & Fortunato, 2009), especially compared to more traditional methods (Depaoli, 2013; Grimm et al., 2017; Harring & Hodis, 2016; Miettunen et al., 2016). Furthermore, many seemingly arbitrary choices need to be made in the application of community detection that so far have not been described. This has forced researchers applying these tools to make these choices for themselves. The goal of the present paper is to describe the application of community detection in clinical applications and prevent unmet expectations in establishing subtypes.

Establishing subtypes is one method of conceptualizing the heterogeneity typically encountered in psychiatry (Agelink van Rentergem et al., 2021; Borsboom et al., 2016; Marquand et al., 2016). Heterogeneity between patients who have the same diagnosis is a challenge to research and practice, as it complicates the search for predictors and treatment options, and makes it difficult to determine the prognosis of individual patients (Bzdok & Meyer‐Lindenberg, 2018). Therefore, researchers are investigating whether there are subtypes of classifications—be it depression (Killian et al., 2021; Lamers et al., 2012), social phobia (Furmark et al., 2000) or Parkinson's disease (Van Rooden et al., 2010)—to reduce this unexplained heterogeneity.

Community detection is a method for identifying subgroups within a network (Fortunato & Hric, 2016). Network science (Barabási, 2016) is roughly synonymous with graph theory—although the two stem from different traditions—and is increasingly being adopted in social science as well as neuroscience (Betzel & Bassett, 2017). Neuroscientists use network science with both functional and structural brain data to model the connections between different brain areas (Mijalkov et al., 2017; Oldham & Fornito, 2019). Areas that are frequently concurrently oxygenated have been found to form stable networks, such as the Default Mode Network. Network science is also increasingly used to model the relationships between different variables, such as questionnaires or test scores (Borsboom et al., 2021; McNally, 2016). For example, the structure of psychopathology has been described using networks (Tio et al., 2016; Wigman et al., 2015) and community detection has been used to identify clusters of items or domains (Blanken et al., 2018; Briganti & Linkowski, 2020; Kendler et al., 2020). Network science is broadly applicable and successful because the underlying theory and mathematics remain the same while nodes, and links between nodes, may represent many different things (Barabási, 2016). Nodes may be airports, proteins, websites, or computers, and links between nodes may be flights, protein–protein interactions, hyperlinks, or wires. In the present application, we are defining nodes as people and the links as similarities between people. The goal is to make subgroups of people who are similar to one another, and are relatively dissimilar to people in other subgroups.

There are several reasons why community detection may be preferable to traditional methods of establishing subtypes such as latent profile analysis, hierarchical clustering, and k‐means clustering. First, traditional measures struggle with somewhat arbitrary choices of rules to decide the number of subgroups. In community detection, the algorithm simultaneously determines the number of subtypes while determining who is a member of which subtype. Second, community detection is flexible in the types of similarity that can be included (Gates et al., 2016). Different choices of similarity measures have different theoretical implications, which we will consider further along in this article. Third, since community detection has been under development for decades in adjacent fields, many supporting methods have been developed. For example, there are established methods to assess the robustness and stability of the subtyping solution (Lancichinetti et al., 2011).

Community detection seems an attractive alternative, but its relative novelty also means that little guidance and knowledge is available. In the ADHD literature, there is a growing number of papers that have established the existence of three temperament subtypes using community detection (Blanken et al., 2021; Goh et al., 2020; Karalunas et al., 2014, 2019; Nigg et al., 2020). In general, community detection is often used for subtyping analyses of ADHD and autism samples (Bathelt et al., 2018; Cordova et al., 2020; Deserno et al., 2022; Fair et al., 2014; Feczko et al., 2018; Groenman, et al., 2019; Mostert et al., 2018; Radhoe, Agelink van Rentergem, Torenvliet, et al., 2021), although there are also applications outside these disorders (e.g., Radhoe, Agelink van Rentergem, Kok, et al., 2021; Saliasi et al., 2015). Since this literature using community detection seems to keep growing, and researchers make use of many untested variations, it is important to examine this method closely.

In this article, we describe different methods of community detection, and briefly describe the traditional methods to which we compare these new algorithms. Then, we discuss the choices that have to be made and steps that have to be taken in conducting a subtyping analysis using community detection. Last, we compare the performance of community detection algorithms in recovering subtype structure in a simulation study.

### 1.1 Community detection algorithms

**FIGURE 1:**
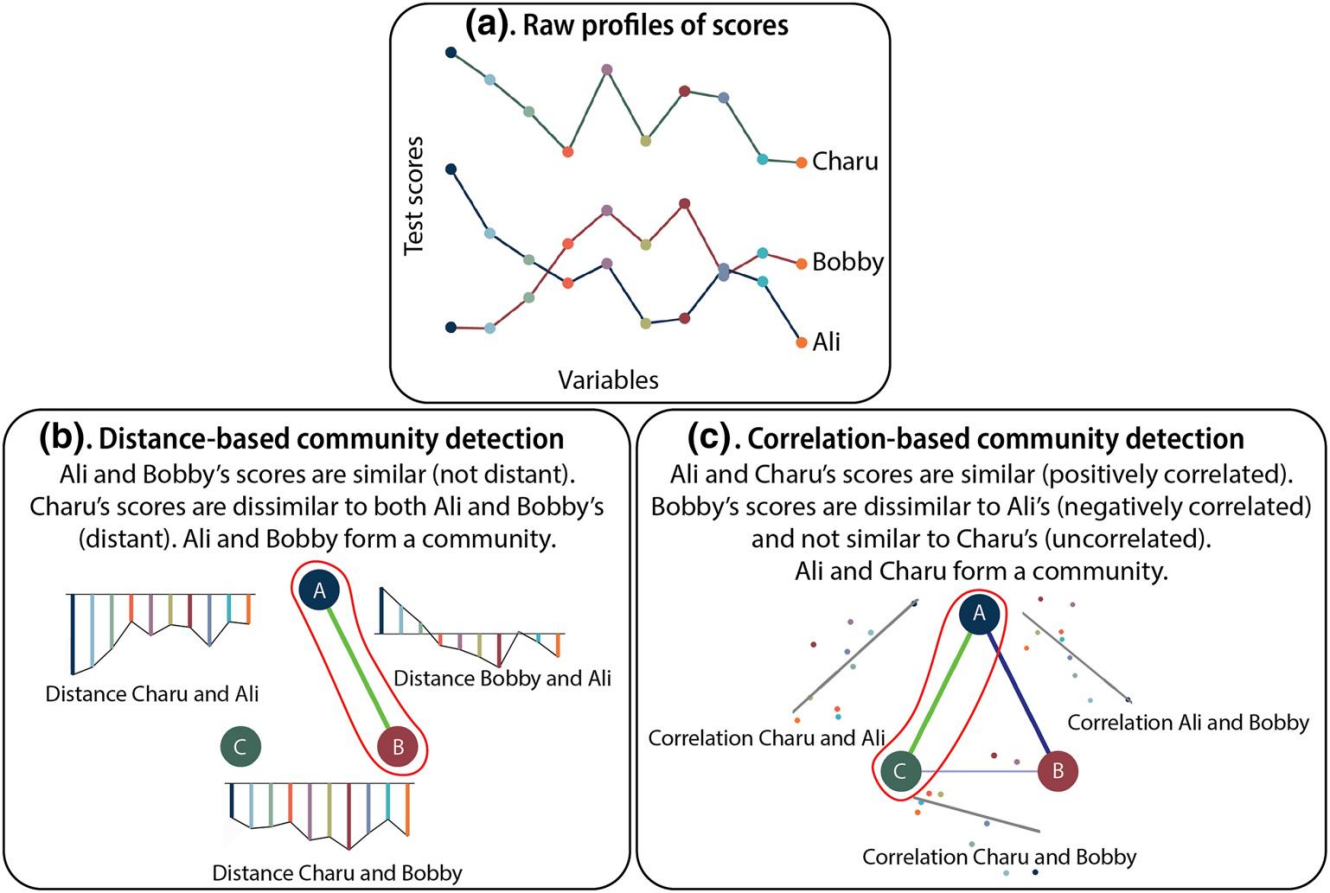
Illustration of how different definitions of similarity can lead to different communities with the same data. In panel (a), example profiles are displayed for three participants (Ali, Bobby, and Charu) who have obtained scores on 10 continuous variables. In panel (b), the distances between participants' scores from panel (a) are displayed for each pair of participants. Note that in practice, multivariate distances would be used, but here all 10 univariate distances are displayed side‐by‐side for illustrative purposes. In panel (c), the correlations between participants' scores from panel (a) are displayed for each pair of participants. In panels (b and c), communities are enclosed in red. Positive similarities are displayed in green, negative similarities in dark blue.

Community detection is a way of finding densely connected communities of nodes within a network. Several community detection algorithms have been developed, and we focus here on three algorithms that are commonly used and have been shown to perform well in previous simulations: Louvain (Blondel et al., 2008), Infomap (Rosvall & Bergstrom, 2008), and Walktrap (Gates et al., 2016; Pons & Latapy, 2005). To explain what community detection does, we will zoom in on the Louvain algorithm. In this algorithm, each node starts off as its own subtype, that is, there are as many subtypes as there are participants. For each participant, we consider the participant leaving its subtype and joining the subtype of one of its neighbors. The decision on whether to join a neighbor and which neighbor to join is based on a quality function, such as modularity (Lancichinetti & Fortunato, 2011), defined as the ratio of links between nodes within the same community to links between nodes within different communities (Fortunato, 2010). The largest increase in the quality function drives the choice. If Ali has some degree of similarity with neighbors Bobby and Charu, and no similarity with David, Erin, Frances, Ali joins either Bobby or Charu based on what leads to the biggest increase in modularity. After that, the next node is considered. If Bobby has some degree of similarity with neighbors Ali, Charu, and Frances, the possibility of Bobby joining one of these neighbors is considered (similarity will later be discussed in more detail in reference to Figure 1). If no increase in modularity is achieved by joining any of these neighbors, Bobby remains alone in his subgroup.

### 1.2 Traditional methods: K‐means and hierarchical clustering, and latent profile analysis

The most popular traditional subgrouping methods—hierarchical clustering, k‐means clustering, and latent profile analyses—are not the focus of this article, but we want to compare the performance of community detection methods to the performance of traditional methods used in psychology. Therefore, we first define what we see as common or reasonable choices for the application of these traditional methods.

K‐means clustering is a popular method in psychiatry (e.g. Adler et al., 2017; Lecavalier, 2006), and is a non‐parametric method meaning that few assumptions need to be made, and is particularly known for its speed. As a decision criterion for the number of subtypes, we will use the Calinski‐Harabasz index (Caliński & Harabasz, 1974) for our comparison. Hierarchical clustering is another popular non‐parametric method of clustering. It is closely related to the taxonomic strategies that are employed in zoology to categorize animals into species, and species into genera (Sneath & Sokal, 1962), but is also frequently used in psychiatric research (e.g., Katsuki et al., 2020). In Ward's method, the squared Euclidean distances between each pair of participants are first calculated. Consecutively, the two participants with the lowest squared Euclidean distance between them are merged, averaging their scores. This is done repeatedly until a single participant remains. The resulting tree is then cut to arrive at a number of subtypes, optimizing an index like we do for the k‐means case.

In contrast with k‐means clustering and hierarchical clustering, latent profile analysis is a parametric method, meaning that a number of assumptions need to be specified regarding the distribution of the data (Magidson & Vermunt, 2002). Because the shape of the assumed distribution is known, inferences can be made on the fit of the model to the data. The distributional assumptions also allow us to assign probabilities of subgroup membership, while other methods assign people to just one subgroup (hard clustering). Moreover, the distributional assumptions allow us to make formal comparisons between models. Different model specifications may consist of different numbers of latent subtypes, different assumed distributions, or different parameter restrictions between subtypes. In this article, we will use the Bayesian Information Criterion (Fraley & Raftery, 1998) for model comparisons, which represents the likelihood of the data given the model, while penalizing the inclusion of extra parameters. For multiple continuous variables, a multivariate normal distribution is typically assumed, which we will also consider throughout this article.

### 1.3 Similarities: Reflected distances and correlation coefficients

In this article, we consider two types of measures of similarity; distance measures of dissimilarity that can be inverted to create measures of similarity, and correlation coefficients. Distance measures such as the Euclidean distance allow us to consider absolute differences between people in scores. For example, if Ali scored 95 on verbal ability, the distance between their 95 and Bobby's 90 is small, and they are more similar to each other than to Charu's 140 (see Figure 1b for a more extensive multivariate example). Euclidean distances are sensitive to mean differences.

Correlation coefficients indicate how profiles of two cases align. If Ali's verbal ability score is their highest out of all their scores, and Charu's verbal ability is also their highest, this indicates that Ali and Charu are similar in their profile, and dissimilar to Bobby, whose highest score is on motor ability. With correlation as a measure of similarity, the absolute value of the scores is not considered. Charu's verbal ability may be 140 and Ali's may be 90. Only the profile of scores is taken into account, that is, strengths and weaknesses are defined only in relation to other strengths and weaknesses (see Figure 1c).

The choice for Euclidean distances or correlations is meaningful for theory and has many practical consequences. To use the example of verbal and non‐verbal IQ, if the researcher is interested in finding out whether there is a subtype of a disorder that is cognitively impaired (Solé et al., 2011), subtypes that reflect mean differences in IQ may be preferred. In this case, similarities may be best characterized as Euclidean distances. If the researcher is interested whether a clinical condition is accompanied by a discrepancy in verbal and non‐verbal IQ (Charman et al., 2011), subtypes that reflect profile differences may be preferred. In this case, similarities may be best characterized as correlations. However, the theoretical implications of the choice of similarity are rarely considered.

If correlations are chosen, a decision has to be taken how to deal with negative correlations, which also has theoretical and practical implications. One has to decide on the basis of theory whether it is desirable for subgroups to be defined in a negative sense, which would mean that participants being unlike other participants means they belong to different subgroups. Practically, negative weights are not implemented for many community detection algorithms (but this is an area of active development, e.g., Traag & Bruggeman, 2009). For these reasons, it is common practice to set all negative weights to 0, thereby losing the information on negative correlations.

Community detection outside of psychiatry traditionally dealt with unweighted links, that are binary: either present or absent (Hoffman et al., 2018), unlike correlations or distances. Unweighted links make community detection simpler, because one can count how many connections there are between nodes within communities, and between nodes across communities. It is possible to define thresholds to dichotomize similarities into present and absent, but this loss of information is generally undesirable. Therefore, dichotomized/binary similarities are not considered in the rest of this article.

## 2 METHODS

**FIGURE 2:**
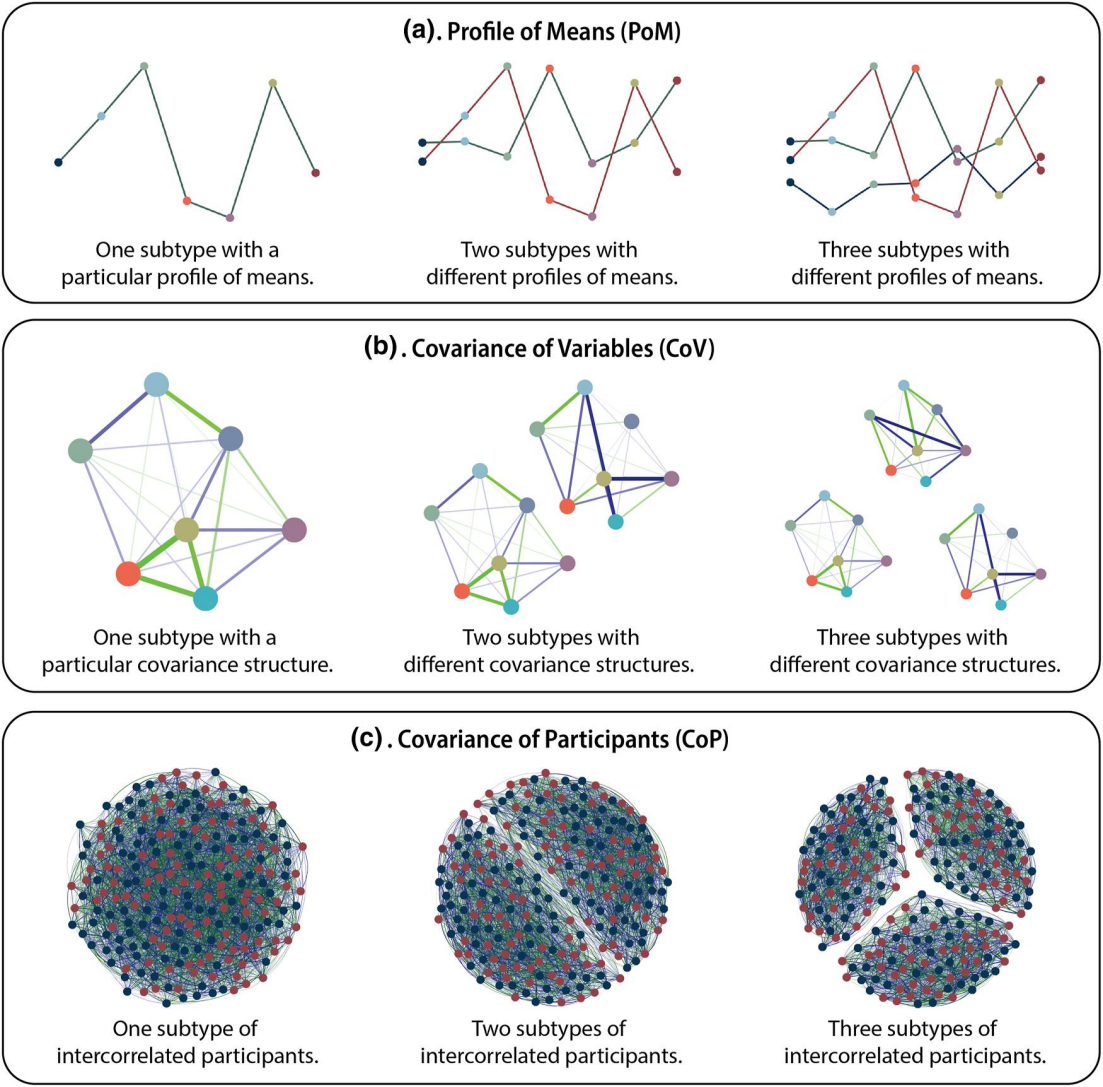
Illustration of the three types of simulated data structure. In panels (a and b), the points refer to different variables; in panel (c) the points refer to participants. In panel (a), the lines between points are illustrative, to show that points are from the same subtype. In panel (b), the lines denote correlations between variables. In panel (c), the lines denote correlations between participants. Positive correlations are displayed in green, negative correlations in dark blue.

To compare community detection results to results of traditional methods, we simulated data for 1, 2, 3, 4 or 5 true subtypes, for a sample size of *N* = 100, *N* = 200, or *N* = 400, with a number of variables (*p*) of 7 or 15. We recently found *N* = 190 to be the median sample size in a sample of 156 subtyping articles in autism, and *p* = 8 to be the median number of variables (Agelink van Rentergem et al., 2021; 82% of articles recovered 2–4 subtypes). Each simulation was repeated 1000 times. The simulation code is provided on OSF (bit.ly/3yjaIqG). Any simulation study that compares methods may be inherently biased towards a particular method. If data is simulated in accordance with particular parametric assumptions, this provides an advantage to parametric methods that make those assumptions. In an effort to avoid this, we simulated data from three different theoretical starting points. Each of these starting points is congruent with a different research tradition. These are illustrated in Figure 2.

In the first approach—which we refer to as the “Profile of Means” (PoM) approach—the basis for the subtypes was the difference between subtypes in mean scores. This is most in line with a “line plot” interpretation typical in the reporting of latent profile analyses, as in Figure 2a. This is a task that is normally completed with other methods but may be more efficiently completed by community detection algorithms. Latent profile analysis, k‐means clustering, and hierarchical clustering should all be able to pick up on differences in means between subtypes. Whether community detection algorithms are sensitive to these differences is not yet known.

In the second approach—which we refer to as the “Correlation of Variables” (CoV)—the basis for the subtypes was the correlational structure of test variables. The mean structure was kept the same across subtypes. This is most in line with a network approach, as in Figure 2b, wherein the correlation between variables is of particular interest rather than mean differences between people (Epskamp et al., 2018). Hierarchical clustering and k‐means clustering should not be able to pick up the differences between correlation structures, as these methods are more dependent on mean structure, while latent profile analyses might, depending on how constrained the chosen model is. Whether community detection algorithms are sensitive to these differences is not yet known.

In the third approach—which we will refer to as the “Correlation of Participants” (CoP)—the basis were the positive correlations between participants within the same subtype, see Figure 2c. This is uncommon in typical simulation studies of psychiatric data but corresponds to the method followed in articles studying the properties of community detection outside of psychiatry (Lancichinetti et al., 2008). For the CoP approach, participant‐level data are still used, even though the community detection algorithms could be fitted to the correlation matrices directly. Simulating participant‐level data ensures that realistic error is still present in the data, and that the same data can be used to compare the community detection results to traditional subgrouping methods. Because this style of simulation is new and catered towards correlation‐based community detection, performance of traditional methods is unpredictable, while community detection based on correlations between participants should perform well.

For each of the simulations, a multivariate normal distribution is used to simulate data. This could potentially bias the results towards traditional methods which assume such distributions—especially latent profile analysis, but as far as we know, there are no other multivariate distributions that are either more psychologically plausible or more in line with the assumptions of community detection algorithms.

The following R‐packages are used: parSim (Epskamp, 2020, for parallelization of simulations), clusterGeneration (Qiu & Joe, 2006, for generating positive definitive covariance matrices), MASS (Venables & Ripley, 2013, for generating multivariate normal data), NbClust (Charrad et al., 2014, for performing k‐means and hierarchical clustering), mclust (Scrucca et al., 2016, for performing latent profile analysis), and igraph (Csardi & Nepusz, 2006, for performing community detection).

## 3 RESULTS

**FIGURE 3:**
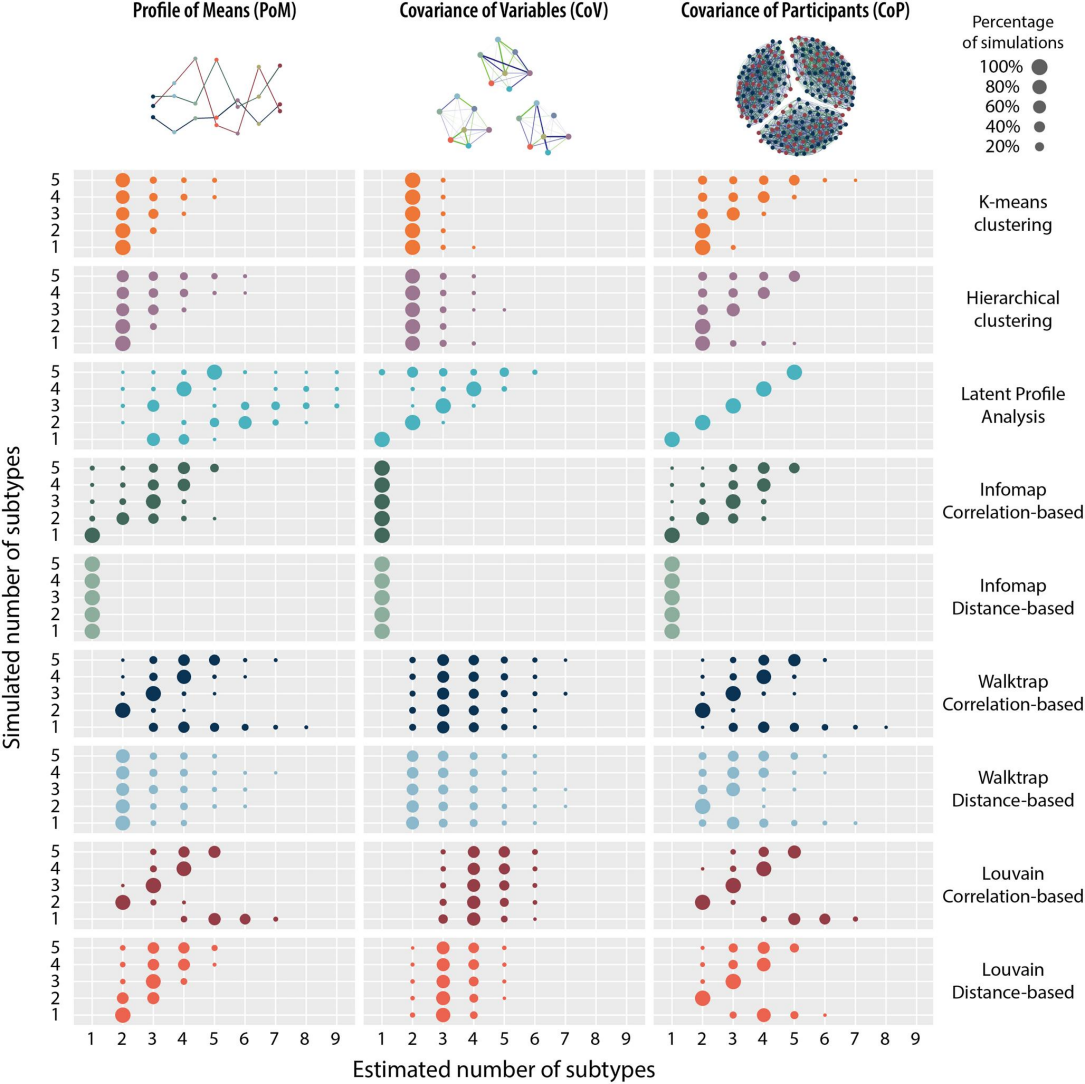
Simulation results. For each true number of subtypes on the *y*‐axis, the distribution over the recovered number of subtypes on the *x*‐axis is plotted. The size of the dots indicates the percentage of the 1000 simulations where that number of subtypes was recovered. If recovery is perfect, all five dots are on the diagonal on the left of the plot, as in the latent profile analysis/covariance of participants panel.

The results presented here are for *p* = 15, and *N* = 400, and are provided in Figure 3. The results for *p* = 7, and *N* = 100 and *N* = 200 are summarized below, and full graphical results for these conditions are provided in Supplementary Materials. As mentioned in the methods section, the simulation code is provided on OSF (bit.ly/3yjaIqG).

### 3.1 PoM: None are perfect, but correlation‐based community detection performs best

For PoM data, the results are provided in the left panels of Figure 3. Community detection algorithms with correlations perform relatively well, although none gets all five of the true numbers of subtypes correct. Louvain and Walktrap get the recovery of one true subtype wrong, but perform well with the rest. Infomap underestimates five subtypes, but performs well with the rest. Latent profile analysis gets better with three to five true subtypes, but dramatically overestimates the number of subtypes when the number of true subtypes is one or two.

The distance‐based community detection algorithms perform worse. Louvain only gets three subtypes correct, and underestimates or overestimates the remainder by one subtype. Walktrap—and k‐means and hierarchical clustering—tends to find two subtypes independent of the true number of subtypes. Infomap always concludes there is one subtype.

### 3.2 Covariance of Variables: Latent profile analysis gives the only reasonable outcome

For CoV data, the results are presented in the middle column of Figure 3. Community detection algorithms with correlations measures perform badly. Louvain tends to conclude there are four subtypes. Walktrap tends to conclude there are two. Infomap consistently finds one. The same is true for distance‐based community detection. With distance‐based measures, Louvain and Walktrap tend to find three subtypes, while Infomap consistently finds one. Hierarchical and k‐means clustering methods find two subtypes. Latent profile analysis tracks the true number of subtypes well for one to four subtypes, and then becomes more volatile for five true subtypes.

### 3.3 Covariance of Participants: Latent profile analysis performs perfectly, others are struggling

For CoP data, the results are presented in the right column of Figure 3. Latent profile analysis tracks the true number of subtypes perfectly. Community detection algorithms with correlations perform relatively well, although none gets all five of the true numbers of subtypes correct. Louvain and Walktrap perform badly with one true subtype but do well with two to four subtypes; five subtypes is somewhat worse again. Infomap does well with one to four subtypes, but underestimates five subtypes.

With distance‐based community detection, Louvain does badly with one true subtype, and well with two to four subtypes; and, again, is worse with five subtypes. Walktrap does badly by tending to inappropriately find two to three subtypes. Infomap always finds one subtype. K‐means and hierarchical clustering find two subtypes when there is one but do better with two to five subtypes.

### 3.4 Community detection affected by lower number of variables, but not sample size

With seven variables instead of 15, all methods are affected to the point where only latent profile analysis provides appropriate results, see Figures S1–S3. With *N* = 200 or *N* = 100, latent profile analysis with the CoP data structure recovers the correct number of subgroups; all other combinations of methods and structures fail to provide meaningful results.

With 15 variables but lower *N*, latent profile analysis gives worse results than for the *N* = 400 situation of Figure 3. However, the community detection results are less affected. The results for Walktrap, Infomap and Louvain are identical to those of the *N* = 400 condition with both *N* = 200 and *N* = 100. See Figures S4 and S5.

## 4 DISCUSSION

In this analysis, we aimed to compare the ability of different community detection and clustering algorithms to recover the true number of subtypes under different simulated conditions. Many of the community detection algorithms applied to our simulated data resulted in subtypes that were wrong. For most settings, latent profile analysis performed as well or outperformed community detection methods in recovering the true structure. Distance‐based community detection did not perform well for any of the three types of data structure that we simulated.

In this article, we have described the theoretical assumptions that go into applying community detection. These choices are not so much due to uncertainty about what the best procedure is, but are more specifically relevant to the researcher's hypotheses regarding the underlying structure of the clinical data. Each statistical method comes with its own assumptions, but when there are default options available that are embedded in commonly‐used software packages, these assumptions are no longer visible to the end user. In that sense, community detection still benefits from its novelty, because the user is still forced to make conscious choices. However, it is difficult to know what data structure is currently being assumed by clinical researchers using community detection. We hope that this article will prompt researchers to be more explicit about their theoretical framework.

Performance of community detection methods suffered when the number of variables was reduced to seven; which is a typical number of variables in applications of community detection in psychiatry (e.g., Fair et al., 2012; Groenman et al., 2019; Mostert et al., 2018). Therefore, the results suggests that this method should be used only with higher numbers of variables. It may specifically be useful for studies with few participants, as community detection gave more stable results for many low *N* situations (i.e., *N* < 400) than traditional methods.

Community detection is evolving as a method, so we were not able to replicate every variation reported in the literature. One study for example, used the intra‐class correlation instead of the correlation as a measure of similarity (Saliasi et al., 2015). Another recent proposal has been to use the output of a random forest prediction to determine similarity, that is, whether two participants often end up in the same leaf in different trees (Feczko et al., 2018). It is an open question whether the (imperfect) results for the community detection algorithms from this article generalize to these other definitions of similarity. One reason that these forms of similarity were not included here is that it becomes quite difficult to imagine what the data‐generating mechanism is for these more elaborate procedures. It has to be noted that for our simulations we used a data‐generating mechanism that resulted in a multivariate normal distribution, which might have biased our results towards better performance for latent profile analysis. Multivariate normality is a common assumption in this type of research (Leese & Landau, 2006; Lubke & Miller, 2015).

Even though some methods performed better overall than others, there was not a single method that performed perfectly in all scenarios. As we have previously advised, we suggest that clinical researchers perform multiple methods to assess the stability of results across methods, as this is not a time‐intensive step and can provide additional evidence on the stability of the results (Agelink van Rentergem et al., 2021). There are already some examples of this in the literature (Karalunas et al., 2019). There are also formal methods to calculate the consensus solution across multiple subtyping methods (Lancichinetti & Fortunato, 2012). Because the results were so variable, it seems best to not overly rely on a single method.

**FIGURE 4:**
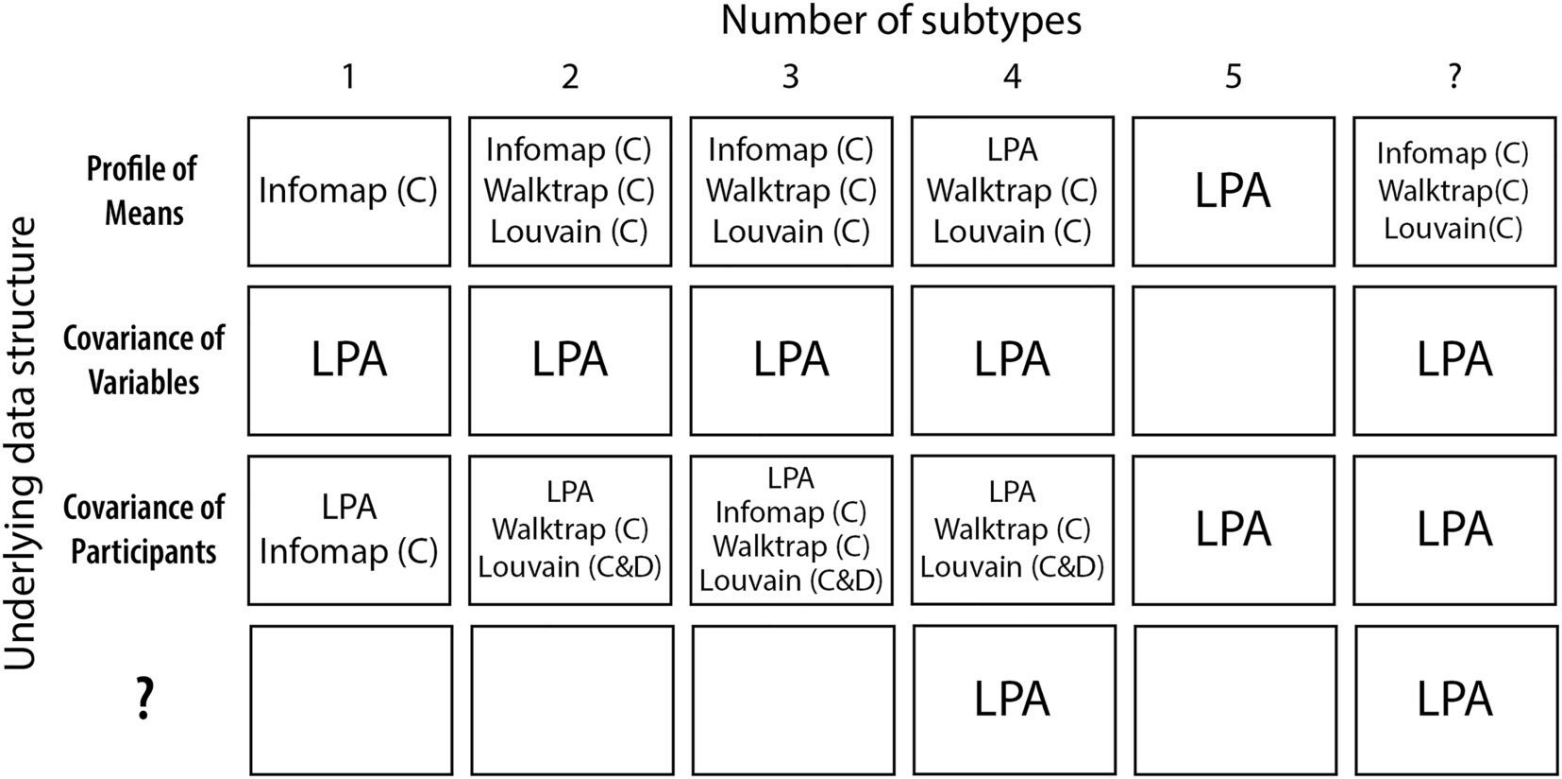
Recommended methods as derived from the simulated datasets. C, correlation‐based; D, distance‐based; LPA, latent profile analysis

To conclude, it is important to realize that community detection algorithms are not created equal, and that results are dependent on the seemingly arbitrary choice of an algorithm. In this article, we have described several analytical choices that clinical researchers have to make based on theoretical and practical considerations. To provide further practical advice for future users, we provide best‐guess recommended methods in Figure 4 based on our simulation results. Most strikingly, we conclude from our results that some algorithms consistently give one particular wrong result. We hope that this result will aid clinical researchers in critically evaluating the results of subgrouping studies. In some ways, the application of community detection to real data may be considered premature, and not retiring traditional methods like latent profile analysis immediately, and comparing results across methods instead of relying on a single method, seems wise.

## AUTHOR CONTRIBUTIONS

**Joost A. Agelink van Rentergem**: Conceptualization; Formal analysis; Investigation; Methodology; Project administration; Visualization; Writing – original draft. **Joe Bathelt**: Code checking; Methodology; Validation; Visualization; Writing – review & editing.**Hilde M. Geurts**: Conceptualization; Funding acquisition; Supervision; Validation; Writing – review & editing.

## CONFLICT OF INTEREST

The authors have no conflict of interest to declare.

## Supporting information

Supporting Information S1

## Data Availability

The simulation code is provided on OSF (bit.ly/3yjaIqG).

## ENDNOTE

^1^
In this article, we use the terms subtype, subgroup, community and cluster interchangeably. These terms are respectively most common in the literature on psychiatry, psychology, community detection, and classification.
