# Supplementary material for: Clinical subtyping using community detection: Limited utility?
Source: Int J Methods Psychiatr Res. 2022 Nov 22;32(2):e1951. doi: 10.1002/mpr.1951 (PMC10242199; doi:10.1002/mpr.1951)
Supplement: Supplementary file 1 — Supporting Information S1 [file MPR-32-e1951-s001.docx]

Supplemental Material: Versions of Figure 3 with lower N, lower p


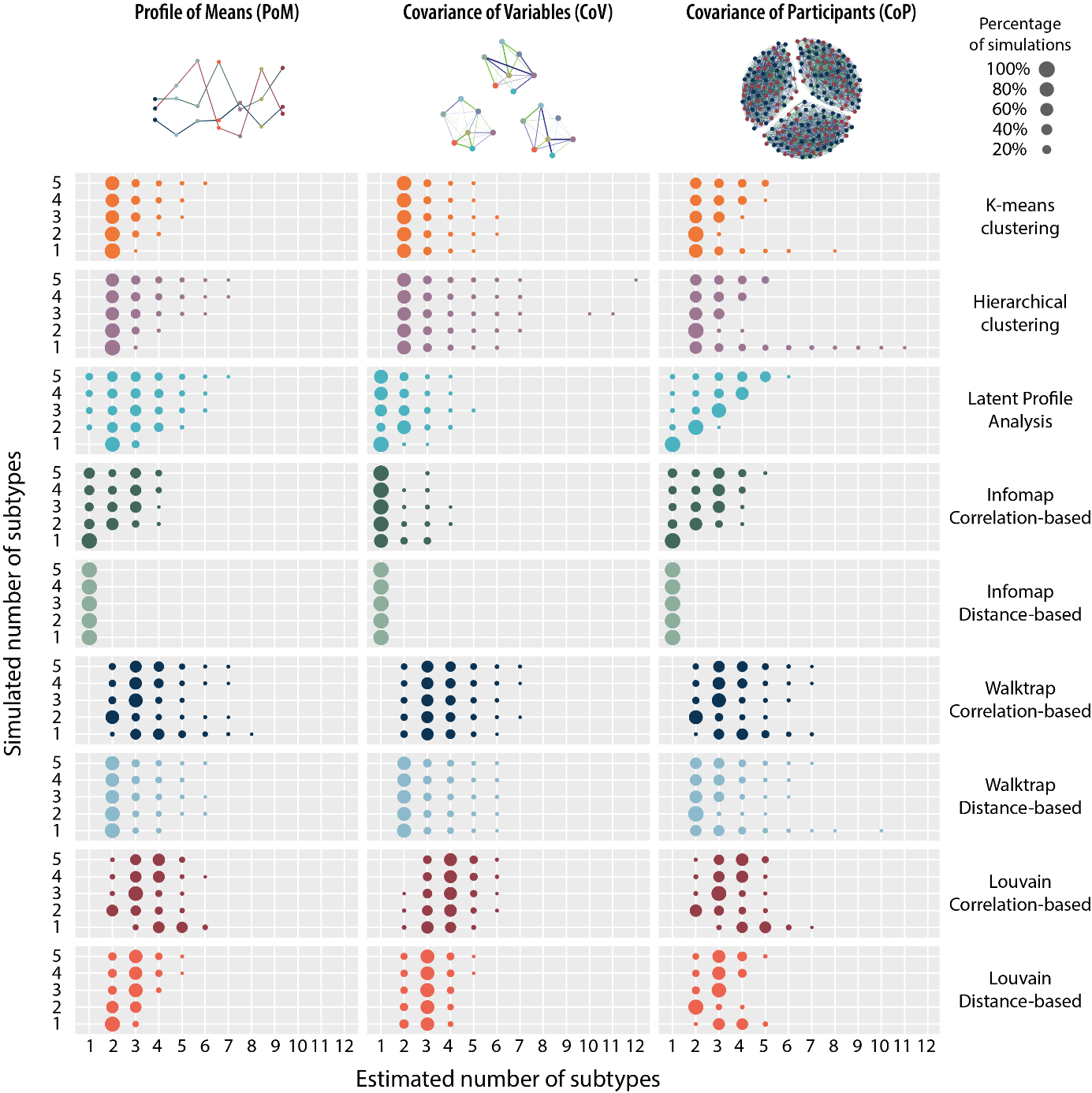


**Figure S1. Number of variables = 7, N = 100**


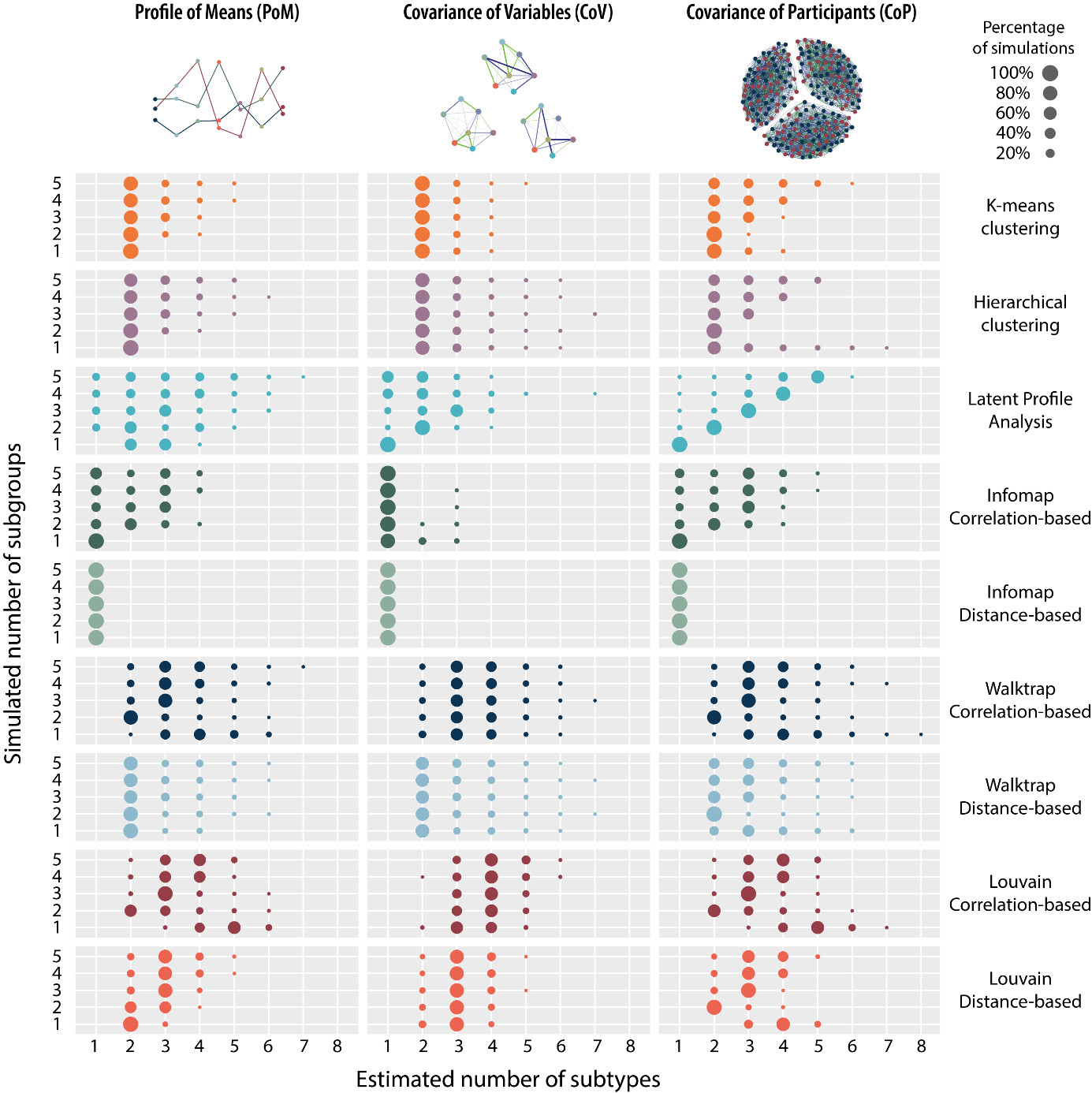


**Figure S2. Number of variables = 7, N = 200**


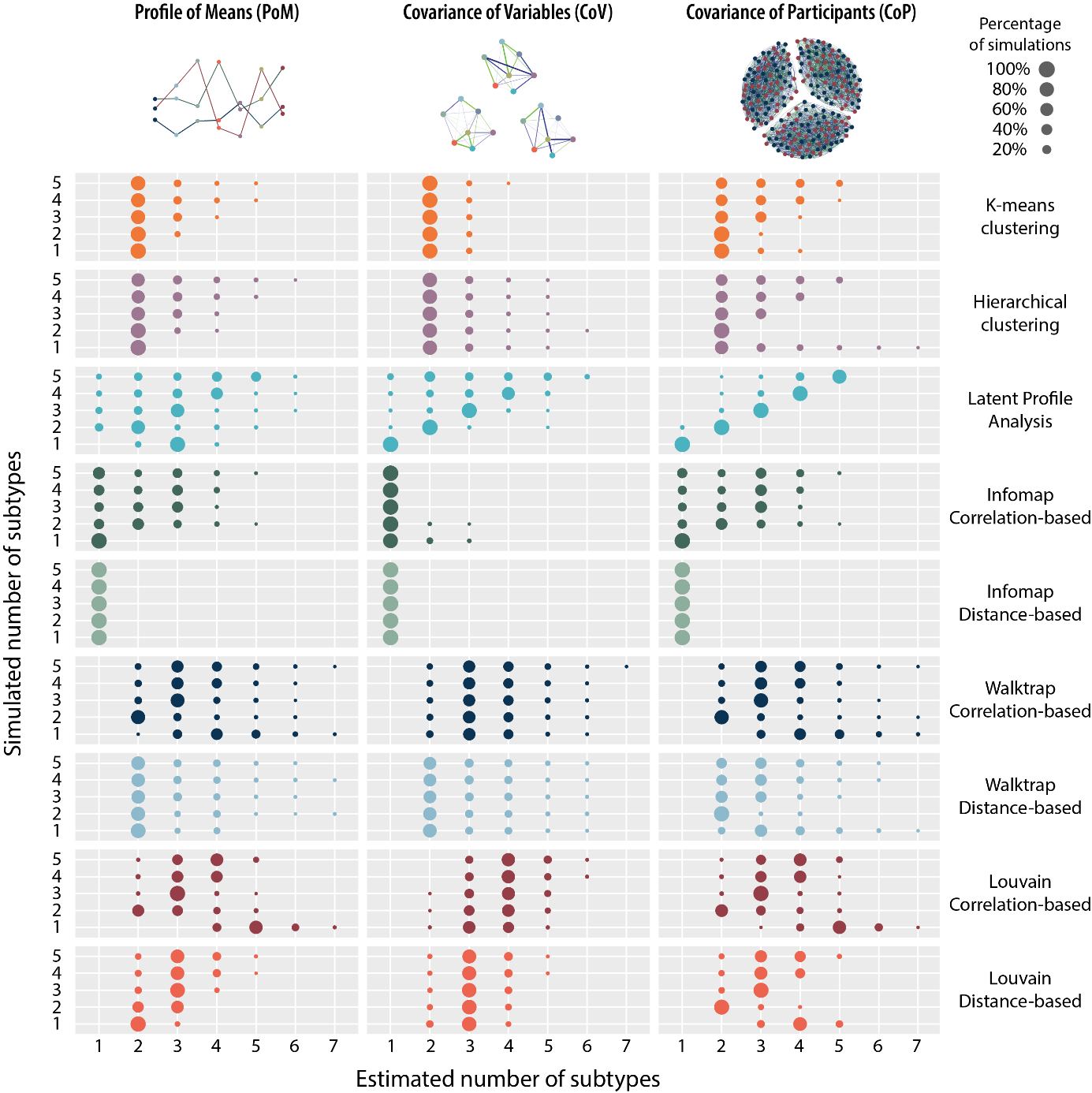


**Figure S3. Number of variables = 7, N = 400**


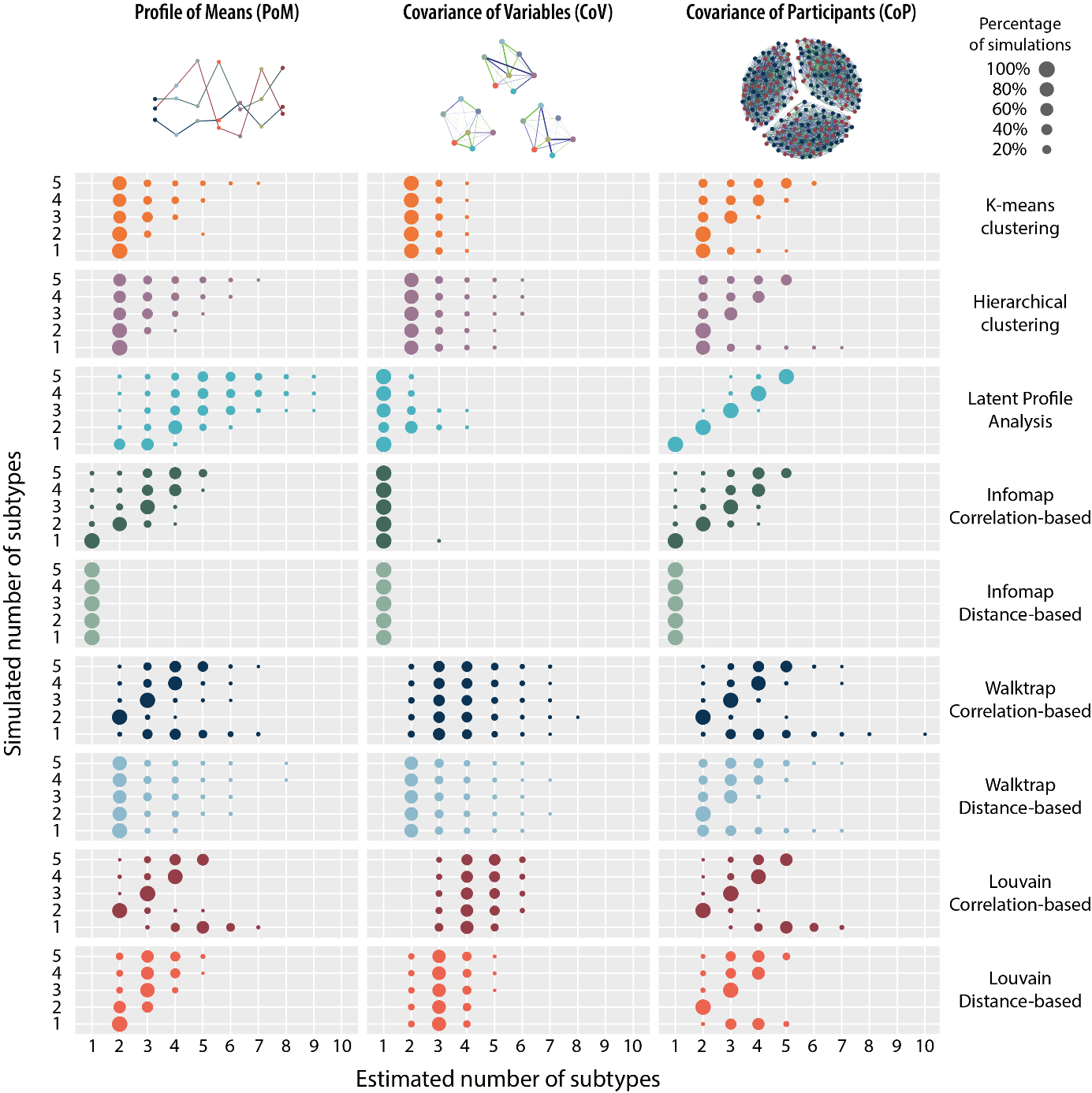


**Figure S4. Number of variables = 15, N = 100**


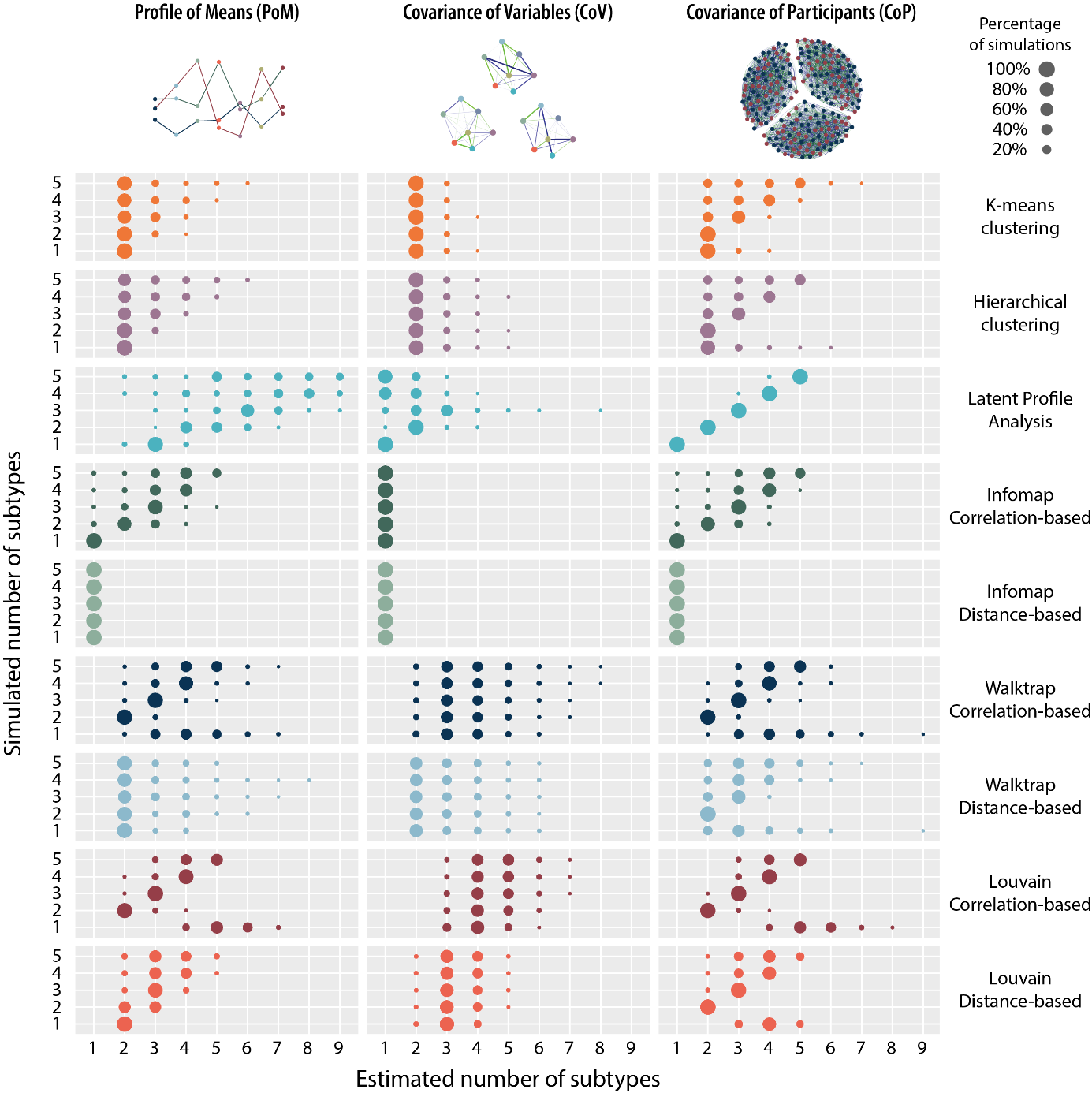


**Figure S5. Number of variables = 15, N = 200**
